# Cornelian Cherry (*Cornus mas* L.) Iridoid and Anthocyanin Extract Enhances PPAR-α, PPAR-γ Expression and Reduces I/M Ratio in Aorta, Increases LXR-α Expression and Alters Adipokines and Triglycerides Levels in Cholesterol-Rich Diet Rabbit Model

**DOI:** 10.3390/nu13103621

**Published:** 2021-10-16

**Authors:** Maciej Danielewski, Alicja Z. Kucharska, Agnieszka Matuszewska, Andrzej Rapak, Agnieszka Gomułkiewicz, Stanisław Dzimira, Piotr Dzięgiel, Beata Nowak, Małgorzata Trocha, Jan Magdalan, Narcyz Piórecki, Adam Szeląg, Tomasz Sozański

**Affiliations:** 1 Department of Pharmacology, Wroclaw Medical University, J. Mikulicza-Radeckiego 2, 50-345 Wroclaw, Poland; agnieszka.matuszewska@umed.wroc.pl (A.M.); beata.nowak@umed.wroc.pl (B.N.); malgorzata.trocha@umed.wroc.pl (M.T.); jan.magdalan@umed.wroc.pl (J.M.); adam.szelag@umed.wroc.pl (A.S.); tomasz.sozanski@umed.wroc.pl (T.S.); 2 Department of Fruit, Vegetable, and Plant Nutraceutical Technology, Wrocław University of Environmental and Life Sciences, J. Chełmonskiego 37, 51-630 Wrocław, Poland; alicja.kucharska@upwr.edu.pl; 3 Hirszfeld Institute of Immunology and Experimental Therapy, Polish Academy of Sciences, R. Weigla 12, 53-114 Wroclaw, Poland; andrzej.rapak@hirszfeld.pl; 4 Department of Human Morphology and Embryology, Wroclaw Medical University, T. Chalubinskiego 6a, 50-368 Wroclaw, Poland; agnieszka.gomulkiewicz@umed.wroc.pl (A.G.); piotr.dziegiel@umed.wroc.pl (P.D.); 5 Department of Pathology, Wrocław University of Environmental and Life Sciences, C. K. Norwida 31, 50-375 Wrocław, Poland; stanislaw.dzimira@upwr.edu.pl; 6 Bolestraszyce Arboretum and Institute of Physiography, Bolestraszyce 130, 37-722 Wyszatyce, Poland; npiorecki@ur.edu.pl; 7 Institute of Physical Culture Sciences, Medical College, University of Rzeszów, Towarnickiego 3, 35-959 Rzeszów, Poland

**Keywords:** cornelian cherry, iridoids, anthocyanins, transcription factors, adipokines, triglycerides, aorta, atherosclerosis, metabolic syndrome

## Abstract

Cornelian cherry (*Cornus mas* L.) fruits possess potential cardiovascular, lipid-lowering and hypoglycemic bioactivities. The aim of this study is to evaluate the influence of resin-purified cornelian cherry extract rich in iridoids and anthocyanins on several transcription factors, intima/media ratio in aorta and serum parameters, which determine or are valuable indicators of the adverse changes observed in the course of atherosclerosis, cardiovascular disease, and metabolic syndrome. For this purpose, male New Zealand rabbits were fed a diet enriched in 1% cholesterol for 60 days. Additionally, one group received 10 mg/kg b.w. of cornelian cherry extract and the second group 50 mg/kg b.w. of cornelian cherry extract. PPAR-α and PPAR-γ expression in the aorta, LXR-α expression in the liver; cholesterol, triglycerides, adipokines, apolipoproteins, glucose and insulin levels in serum; the intima and media diameter in the thoracic and abdominal aorta were determined. Administration of cornelian cherry extract resulted in an enhancement in the expression of all tested transcription factors, a decrease in triglycerides, leptin and resistin, and an increase in adiponectin levels. In addition, a significant reduction in the I/M ratio was observed for both the thoracic and abdominal aorta. The results we have obtained confirm the potential contribution of cornelian cherry extract to mitigation of the risk of developing and the intensity of symptoms of obesity-related cardiovascular diseases and metabolic disorders such as atherosclerosis or metabolic syndrome.

## 1. Introduction

There are many groups of plant compounds with proven curative effects on the body. These include, among others, iridoids and anthocyanins. Both groups affect positively especially the function of the cardiovascular system and the liver. Up to this date cardiovascular, hypoglycemic, hypolipidemic, antihepatotoxic, choleretic, anti-inflammatory, antispasmodic, antitumor, antiviral, immunomodulatory and purgative activities of iridoids [1,2] and anti-oxidative, antimicrobial, anti-inflammatory, anti-aging, lipid-lowering, anti-diabetic, anti-cancerous, and anti-obesity activities of anthocyanins were reported [3,4,5,6].

One of the valuable sources of both iridoids and anthocyanins is the cornelian cherry fruits. *Cornus mas* L., from the *Cornaceae* family, is a branchy shrub or small tree, native to central and south-eastern Europe and western Asia. In addition to iridoids and anthocyanins, cornelian cherry fruits contain also other flavonoids (e.g., flavonols), phenolic acids, terpenoids (ursolic acid), carotenoids and organic acids [7,8,9,10]. Due to the significant content of the iridoids and anthocyanins, cornelian cherry fruits may play an important role in the prevention and treatment of many diseases of the cardiovascular system and the liver [11,12].

The excess level of cholesterol, often resulting from improper diet and lack of adequate physical activity contributes to the development of hyperlipidemia and then possible atherosclerosis, metabolic syndrome, hypertension or cardiovascular disease (CVD) [13,14,15,16]. Cholesterol overabundance, broaden by frequently accompanying chronic inflammation, can also lead to aortic dysfunctions, which in extreme cases may result in the aortic aneurysm or aortic valve insufficiency [17,18]. Noticeable alterations in the levels of compounds important for lipid metabolism and transport, e.g., adipokines, as well as changes in the expression of transcription factors, such as peroxisome proliferator-activated receptors (PPARs) and liver X receptors (LXRs) are also observed [19,20,21].

The previous findings [4,22,23,24] showed that cornelian cherry fruits are a prospectively valuable source of chemicals that can play an exploitable role in preventing adverse changes in the cardiovascular system, including the aorta and also the liver. However, the impact of cornelian cherry’s iridoids and anthocyanins on transcription factors that play an important role in lipid and cholesterol metabolism, and also on many serum parameters crucial in the development and diagnostics of cardiovascular and liver diseases, is not fully explored.

The aim of this study is to determine and compare whether and to what extent two different doses of resin-purified cornelian cherry extract (10 mg per kilogram of body weight or 50 mg per kilogram of body weight) have an effect on the expression of peroxisome proliferator-activated receptor-α (PPAR-α), peroxisome proliferator-activated receptor-γ (PPAR-γ) in the aorta, and liver X receptor-α (LXR-α) in the liver; on serum levels of leptin, adiponectin, resistin, glucose, insulin and selected lipoproteins and apolipoproteins (Apos); and on aortal intima/media ratio (histopathological changes) in the rabbit model. We also try to compare changes in blood markers–those that play an important role in clinical practice in predicting and assessing the risk and progression of cardiovascular and metabolic diseases–with molecular and histopathological changes in target organs. So far, to our knowledge, no one has studied these features so thoroughly, and we believe that our results may constitute another important step towards full determination of the therapeutic value of cornelian cherry fruits and their potential use in the prophylaxis and therapy of cardiovascular diseases.

## 2. Materials and Methods

### 2.1. Animal Model 

Fifty sexually mature male New Zealand rabbits aged 8 months to 1 year were used in the experiment. The animals were housed in individual chambers with temperatures maintained at 21–23 °C. Rabbits were acclimated, weighed and observed for four weeks prior to the beginning of the sixty-day study. Then, they were randomly divided into 5 groups of 10 animals. The animals in group P were fed the standard complete formula for rabbits. Animals in other groups: CHOL, EXT 10, EXT 50 and SIMV 5 were fed with the above-mentioned mixture enriched in 1% cholesterol. During the experiment, rabbits had *ad libitum* water access and received the same daily portion of chow (40 g/kg). Once-daily, in the morning, for the consecutive 60 days of the study, the following substances were administered orally to the rabbits: groups P and CHOL–normal saline solution, group EXT 10–*Cornus mas* L. extract 10 mg per kg b.w., group EXT 50–*Cornus mas* L. extract 50 mg per kg b.w., group SIMV 5–simvastatin 5 mg per kg b.w. The feeding schema is presented in Table 1.

Blood samples were collected before and after 60 days of the experiment from each animal, from the margin vein of the ear or the saphenous vein. At the end of a study, the rabbits were euthanized with terminal anesthesia, using Morbital^®^ (Biowet, Puławy, Poland; 1 mL of the drug contains 133.3 mg of sodium pentobarbital and 26.7 mg of pentobarbital) at a dose of 2 mL/kg given intraperitoneally (i.p.). The aortas and livers were then harvested and cleaned, and afterward frozen and stored at the temperature of −70 °C for further examination.

### 2.2. Chemicals and Materials

Acetonitrile, methanol, and formic acid were purchased from Sigma-Aldrich (Steinheim, Germany). Loganic acid, *p*-coumaric acid, caffeic acid, ellagic acid, quercetin 3-*O*-glucoside, kaempferol 3-*O*-glucoside, and cyanidin 3-*O*-glucoside were purchased from Extrasynthese (Lyon Nord, France). 

### 2.3. Plant Materials and Preparation of Extract

**Figure 1:**
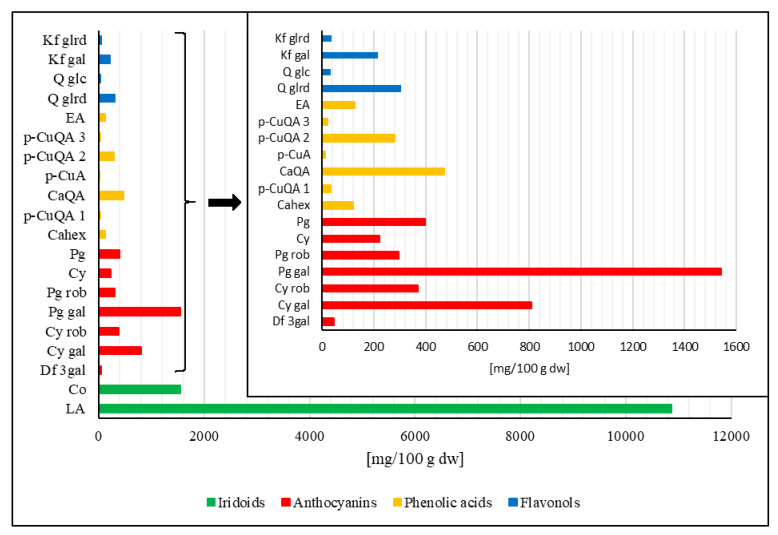
Iridoids and polyphenols (anthocyanins, phenolic acids, and flavonols) content in the purified cornelian cherry extract. Abbreviations: LA–loganic acid, Co–cornuside; Df 3 gal–delphinidin 3-*O*-galactoside; Cy gal–cyanidin 3-*O*-galactoside; Cy rob–cyanidin 3-*O*-robinobioside; Pg gal–pelargonidin 3-*O*-galactoside; Pg rob–pelargonidin 3-*O*-robinobioside; Cy–cyanidin; Pg–pelargonidin; Cahex–caffeoylhexoside; *p*-CuQA 1–*p*-coumaroilquinic acid 1; CaQA–caffeoylquinic acid; *p*-CuA–*p*-coumaric acid; *p*-CuQA 2–*p*-coumaroilquinic acid 2; *p*-CuQA 3–*p*-coumaroilquinic acid 3; EA–ellagic acid; Q glrd–quercetin 3-*O*-glucuronide; Q glc–quercetin 3-*O*-glucoside; Kf gal–kaempferol 3-*O*-galactoside; Kf glrd–kaempferol 3-*O*-glucuronide.

Cornelian cherry (*Cornus mas* L.) fruits were assembled in the Bolestraszyce Arboretum and Institute of Physiography, Poland. Before analysis, fruits were stored in a falcon tube at −20°C. A plant herbarium specimen (BDPA 3 967) was authenticated and deposited in the Herbarium of the Bolestraszyce Arboretum and Institute of Physiography, Poland. The preparation of a resin-purified cornelian cherry extract (EXT) was previously described by Nowak et al. [25]. The quantitative composition of iridoids and polyphenols in the EXT is shown in Figure 1.

### 2.4. Quantification of Compounds by HPLC-PDA

The assay of iridoids and anthocyanins was carried out according to the method described by Kucharska et al. [26], with a Dionex HPLC system (Germering, Germany) equipped with the Ultimate 3000 model diode array detector. The Cadenza Imtakt column CD-C18 (75 × 4.6 mm, 5 µm) was used. The mobile phase was a mixture of 4.5% aq. formic acid, *v*/*v* (A) and 100% acetonitrile (B), flowing at 1.0 mL min − 1 under gradient elution conditions: 0–1 min 5% B in A, 1–20 min 25% B in A, 20–26 min 100% B, 26–30 min 5% B in A. Iridoids were monitored at wavelengths of 245 nm, ellagic acid at 254 nm, phenolic acids at 320 nm, flavonols at 360 nm, and anthocyanins at 520 nm. Iridoids were quantified as loganic acid, anthocyanins as cyanidin 3-*O*-glucoside, phenolic acids as *p*-coumaric acid, caffeic acid, and ellagic acid, flavonols as quercetin 3-*O*-glucoside and kaempferol 3-*O*-glucoside.

### 2.5. Measurement of Physical and Biochemical Parameters

The bodyweight of the rabbits was determined using a precision scale (Radwag, Radom, Poland). The serum levels of total cholesterol (TC) and triglycerides (TGs) were estimated based on calorimetric, enzymatic methods. High-density lipoprotein (HDL-C) and low-density lipoprotein cholesterols (LDL-C) were measured using calorimetric, direct methods (ABX Pentra 400, Horiba Ltd., Kyoto, Japan). Glucose levels were determined using the Trinder glucose activity test (ABX Pentra 400, Horiba Ltd., Kyoto, Japan).

### 2.6. Quantification of Adiponectin, Leptin, Resistin, Apolipoproteins (A1, B100, E), and Insulin by Enzyme-Linked Immunosorbent Assay (ELISA)

ELISA method was used in the determination of adiponectin (ELISA kit for Rabbit ADP/Acrp30, ERB0002, Fine Test, Wuhan Fine Biotech Corp., Wuhan, China), leptin (Rabbit Leptin ELISA Kit, CSB-E06971Rb, Cusabio Technology LLC, Houston, TX, USA), resistin (Rabbit Resistin ELISA kit, ERB0105, Fine Test, Wuhan Fine Biotech Corp., Wuhan, China), apolipoprotein A1 (ELISA kit for Rabbit Apolipoprotein A1, ERB0009, Fine Test, Wuhan Fine Biotech Corp., Wuhan, China), apolipoprotein B100 (ELISA kit for Rabbit Apolipoprotein B100, ERB0010, Fine Test, Wuhan Fine Biotech Corp., Wuhan, China), apolipoprotein E (ELISA kit for Rabbit Apolipoprotein E, ERB0014, Fine Test, Wuhan Fine Biotech Corp., Wuhan, China), and insulin (Rabbit Insulin ELISA kit, 90186, Crystal Chem Inc., Elk Grove Village, IL, USA) levels, according to manufacturer’s instructions. All concentrations were expressed as ng/mL or mmoL/L.

### 2.7. Histopathological Assessment of the Thoracic and Abdominal Aorta

The material fixed in buffered 7% formalin was embedded in paraffin and cut into 4 µm sections which were stained by the routine hematoxylin-eosin method. The preparations were viewed without knowing the division into the control group and experimental groups. Microscopic analysis was performed using the Olympus BX53 light microscope coupled with Olympus UC90 camera. The intima and media thickness measurements were made using a cellSens standard V1 software. Photos were made at an enlargement of 100× or 200×, the scale shown in the photo is 50 µm or 100 µm.

### 2.8. RNA Isolation, Reverse Transcription and Real-Time PCR

Total RNA was isolated from studied tissue samples with RNeasy Fibrous Mini Kit (Qiagen, Hilden, Germany) according to the manufacturer’s protocol. To eliminate genomic DNA contamination, on-column DNase digestion was performed using RNase-Free DNase Set (Qiagen, Hilden, Germany). Quantity and purity of RNA samples were assessed by measuring the absorbance at 260 and 280 nm with NanoDrop1000 spectrophotometer (Thermo Fisher Scientific, Waltham, MA, USA). First-strand cDNA was synthesized using the High Capacity cDNA Reverse Transcription Kit (Applied Biosystems, Carlsbad, CA, USA) as described in the protocol. The mRNA expression of PPAR-α and PPAR-γ was determined by quantitative real-time PCR with 7500 Real-Time PCR System and Power SYBR Green PCR Master Mix (Applied Biosystems, Carlsbad, CA, USA). Glyceraldehyde 3-phosphate dehydrogenase (GAPDH) was used as the reference gene. The reactions were performed with RT² qPCR Primer Assay for Rabbit PPAR-α (PPN13311A, Qiagen, Hilden, Germany), PPAR-γ (PPN05175A, Qiagen, Hilden, Germany), and GAPDH (PPN00377A, Qiagen, Hilden, Germany). All reactions were performed in triplicates under the following conditions: activation of the polymerase at 50 °C for 2 min, initial denaturation at 94 °C for 10 min and 40 cycles of denaturation at 94 °C for 15 s followed by annealing and elongation at 60 °C for 1 min. The specificity of the PCR was determined by melt curve analysis for each reaction. The relative mRNA expression of the examined factors was calculated with the ∆∆Ct method.

### 2.9. Western Blotting of LXR-α Expression

Rabbit livers were homogenized in buffer containing 25 mM Tris pH 7.5, 50 mM NaCl, 1% NP-40 and protease inhibitors set. After centrifugation, the clear supernatant was mixed with the SDS sample buffer, boiled at 95 °C for 5 min and subjected to SDS-PAGE on 12% gel. The resolved proteins were transferred to the PVDF membrane (Thermo Fisher Scientific, Waltham, MA, USA) using semi-dry transfer. After the transfer, the membrane was blocked with 1% casein in TBS at 4 °C, overnight, and then incubated with 1 µg/mL of antibody anti-LXR-α (NR1H3/LXR Alpha Antibody LS-B3526-50, LifeSpan Biosciences Inc., Seattle, WA, USA) and beta-actin C-04 (Santa Cruz Biotechnology Inc., Dallas, TX, USA) at room temperature for 1 h, followed by secondary horseradish peroxidase-labeled antibody (Dako, Agilent, Santa Clara, CA, USA). The bounded antibodies were visualized using the West-Pico blotting detection system (Thermo Fisher Scientific, Waltham, MA, USA). The blots were scanned, and the optical density of bands was analysed with Image J software.

### 2.10. Statistical Analysis

Parametric data were expressed as mean ± standard deviation (mean ± SD). The statistical analysis was conducted using the Statistica v. 13.3 software (TIBCO Software Inc., Palo Alto, CA, USA). The normality of all continuous variables was verified with the Shapiro–Wilk test. One-way analysis of variance (ANOVA) with least significant difference (LSD) Fisher’s post hoc test was performed for a comparison involving 3 or more groups. The *p*-values < 0.05 were considered statistically significant. Graphical representations of the statistical data were created using the Statistica v. 13.3 software (TIBCO Software Inc., Palo Alto, CA, USA).

## 3. Results

We have studied the effects of the oral administration of resin-purified cornelian cherry extract on mRNA expression of PPAR-α and PPAR-γ in the aorta and LXR-α expression in hepatocytes. We have indicated the levels of various parameters in the serum, and we conducted also a histopathological analysis of the intima-media ratio in the thoracic and abdominal aorta.

### 3.1. Body Weight and Serum Lipid Levels

Firstly, we assessed the change in body weight of the rabbits. On day 60 (the last day of the study), we observed an increase in average body weight among all study groups. The highest increase in average body weight was noted in the SIMV 5 group and the CHOL group. The smallest weight gain compared to the control group was recorded in the EXT 50 group. Summary data are presented in Table 2.

**Figure 2:**
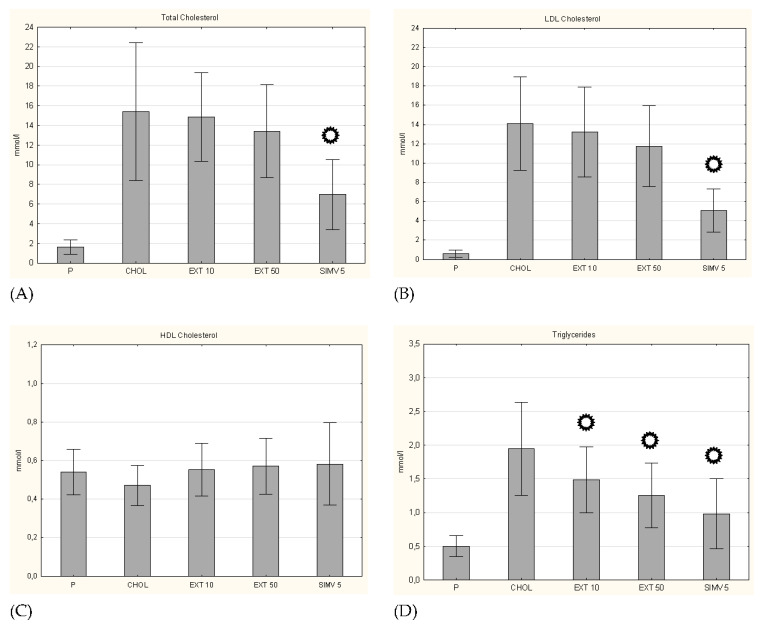
Serum lipid levels. (**A**) total cholesterol, (**B**) LDL cholesterol, (**C**) HDL cholesterol, and (**D**) triglycerides levels on day 60. of the study. P–standard chow; CHOL–standard chow + 1% cholesterol; EXT 10–standard chow + 1% cholesterol + cornelian cherry extract 10 mg/kg b.w.; EXT 50–standard chow + 1% cholesterol + cornelian cherry extract 50 mg/kg b.w.; SIMV 5–standard chow + 1% cholesterol + simvastatin 5 mg/kg b.w. Values are presented as mean ± SD. 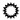 *p* < 0.05 vs. CHOL.

In quantification of serum lipid levels, a significant increase in the total cholesterol level was observed in the CHOL group compared to the P group. In both groups fed with the cholesterol diet with the cornelian cherry extract, a slightly smaller increase in TC levels was observed (compared to the P group), but only in the simvastatin group, the change was statistically significant (*p* < 0.001). In the case of the LDL-C fraction, an identical trend was observed, while in the HDL-C level evaluation the alterations were negligible. On the contrary, the measurement of triglycerides showed significant decreases in their levels in all three research groups (EXT 10 *p* = 0.036; EXT 50 *p* = 0.002; SIMV 5 *p* < 0.001) (Figure 2).

### 3.2. Expression of PPAR-α and PPAR-γ in the Aorta and LXR-α in the Liver

Feeding a cholesterol-rich diet compared to the baseline caused a decrease in the mRNA expression of PPAR-α and PPAR-γ in the aorta, while uptake of cornelian cherry extract led to an increase in the expression of both transcription factors, wherein receiving of 10 mg/kg b.w. extract changed significantly the expression of PPAR-α (*p* = 0.035) and receiving of 50 mg/kg b.w. extract changed significantly the expression of PPAR-γ (*p* = 0.031).

**Figure 3:**
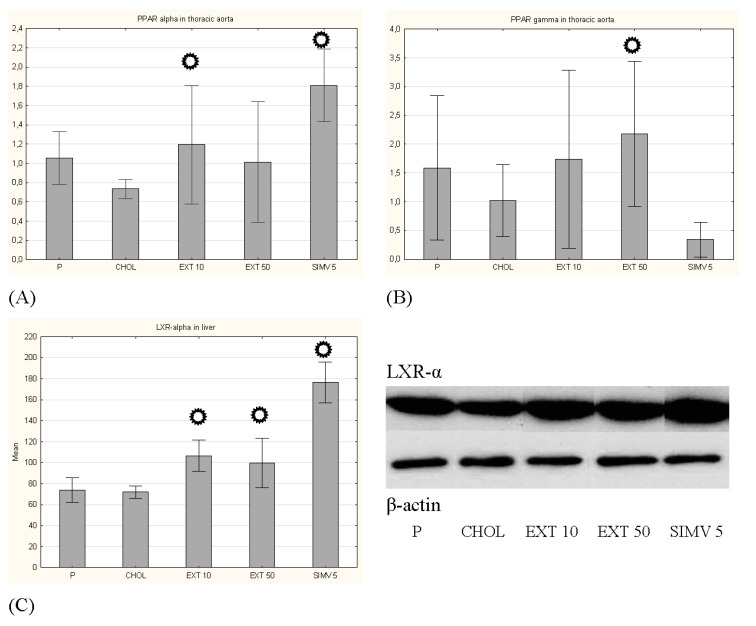
Expression of transcription factors. (**A**) PPAR-α in aorta, (**B**) PPAR-γ in aorta, (**C**) LXR-α in liver. P–standard chow; CHOL–standard chow + 1% cholesterol; EXT 10–standard chow + 1% cholesterol + cornelian cherry extract 10 mg/kg b.w.; EXT 50–standard chow + 1% cholesterol + cornelian cherry extract 50 mg/kg b.w.; SIMV 5–standard chow + 1% cholesterol + simvastatin 5 mg/kg b.w. Values are presented as mean ± SD. 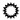 *p* < 0.05 vs. CHOL.

Using the Western blot method with bands optical density assessment, we determined the expression of another transcription factor–liver X receptor alpha–LXR-α. The cholesterol diet slightly decreased, while the consumption of cornelian cherry extract significantly induced the LXR-α expression, with a greater effect observed in the group receiving 10 mg/kg b.w (*p* = 0.001, in comparison to *p* = 0.007 in EXT 50 group), while the greatest augmentation was noted in the SIMV 5 group (*p* < 0.001) (Figure 3).

### 3.3. Adipokines Serum Levels

**Figure 4:**
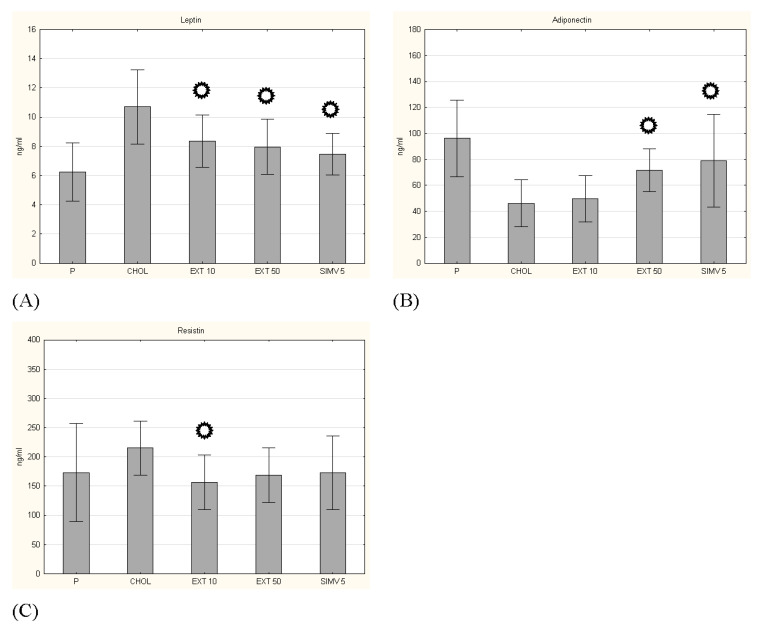
Concentrations of adipokines. (**A**) leptin, (**B**) adiponectin and (**C**) resistin. P–standard chow; CHOL–standard chow + 1% cholesterol; EXT 10–standard chow + 1% cholesterol + cornelian cherry extract 10 mg/kg b.w.; EXT 50–standard chow + 1% cholesterol + cornelian cherry extract 50 mg/kg b.w.; SIMV 5–standard chow + 1% cholesterol + simvastatin 5 mg/kg b.w. Values are presented as mean ± SD. 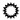 *p* < 0.05 vs. CHOL.

In the case of leptin serum level, a beneficial effect of cornelian cherry extract was observed in both dosing regimens—in both cases, changes were statistically significant (respectively *p* = 0.011 and *p* = 0.003). On the other hand, in the measurement of adiponectin, the beneficial effect of the extract was also observed for both dosing regimens, but only for the amount of 50 mg/kg b.w. the changes were statistically significant (*p* = 0.027). The positive effect of cornelian cherry extract on the serum level of resistin was also confirmed. Administration in the amount of 10 mg/kg b.w. caused a significant change (*p* = 0.032) in the level of resistin compared to the CHOL group (Figure 4).

### 3.4. Apolipoproteins, Glucose and Insulin Levels

**Figure 5:**
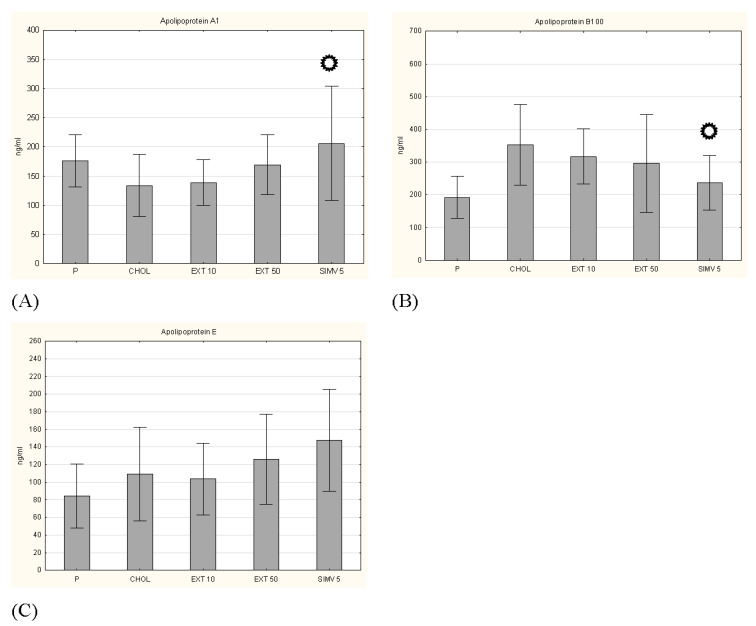
Concentrations of apolipoproteins. (**A**) apolipoprotein A1, (**B**) apolipoprotein B100 and (**C**) apolipoprotein E. P–standard chow; CHOL–standard chow + 1% cholesterol; EXT 10–standard chow + 1% cholesterol + cornelian cherry extract 10 mg/kg b.w.; EXT 50–standard chow + 1% cholesterol + cornelian cherry extract 50 mg/kg b.w.; SIMV 5–standard chow + 1% cholesterol + simvastatin 5 mg/kg b.w. Values are presented as mean ± SD. 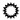 *p* < 0.05 vs. CHOL.

**Figure 6:**
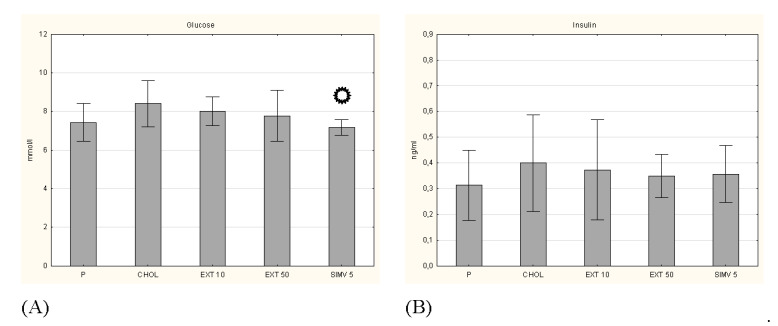
Concentrations of (**A**) glucose and (**B**) insulin. P–standard chow; CHOL–standard chow + 1% cholesterol; EXT 10–standard chow + 1% cholesterol + cornelian cherry extract 10 mg/kg b.w.; EXT 50–standard chow + 1% cholesterol + cornelian cherry extract 50 mg/kg b.w.; SIMV 5–standard chow + 1% cholesterol + simvastatin 5 mg/kg b.w. Values are presented as mean ± SD. 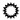 *p* < 0.05 vs. CHOL.

Other investigated parameters were the levels of selected apolipoproteins, i.e., protein fragments of lipoproteins that are responsible for lipid binding. The results for apolipoprotein A1, B100 and E were determined. In the case of Apo A1 and B100, we observed a certain favorable tendency caused by the oral intake of cornelian cherry extract, but the obtained changes did not turn out to be statistically significant (Figure 5). In turn, measurement of Apo E showed opposite effects of two studied doses, a slight decrease in Apo E level by 10 mg/kg b.w. dosage and a slight increase in Apo E level by 50 mg/kg b.w. dosage. Examination of glucose and insulin levels also did not show a significant result (Figure 6), similarly to the measurement of the insulin resistance index (HOMA-IR) (Table 3).

### 3.5. Intima, Media and I/M Ratio in the Thoracic and the Abdominal Aorta

**Figure 7:**
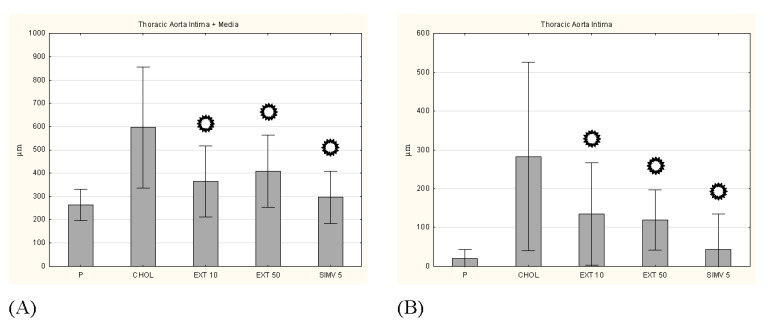

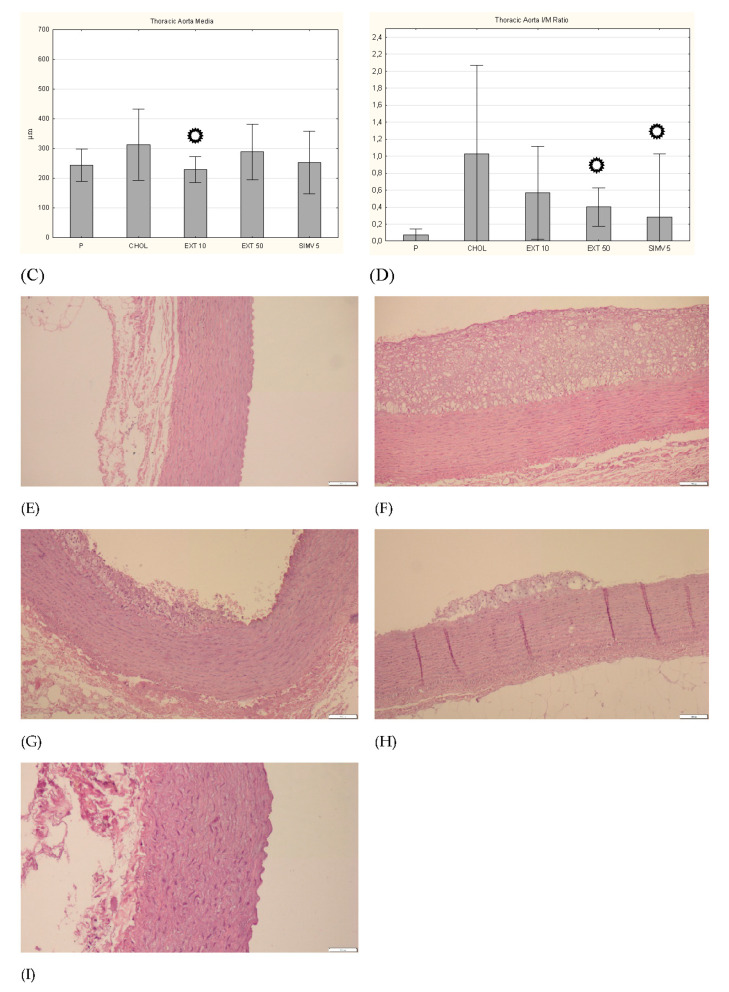
Intima and media thickness, and the intima/media ratio in the thoracic aorta. (**A**) intima + media thickness, (**B**) intima thickness, (**C**) media thickness, and (**D**) I/M ratio. P–standard chow; CHOL–standard chow + 1% cholesterol; EXT 10–standard chow + 1% cholesterol + cornelian cherry extract 10 mg/kg b.w.; EXT 50–standard chow + 1% cholesterol + cornelian cherry extract 50 mg/kg b.w.; SIMV 5–standard chow + 1% cholesterol + simvastatin 5 mg/kg b.w. Values are presented as mean ± SD. 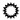 *p* < 0.05 vs. CHOL. Representative cross-sections of the thoracic aorta segments, stained with hematoxylin-eosin in the (**E**) P group, (**F**) CHOL group, (**G**) EXT 10 group, (**H**) EXT 50 group and (**I**) SIMV 5 group.

**Figure 8:**
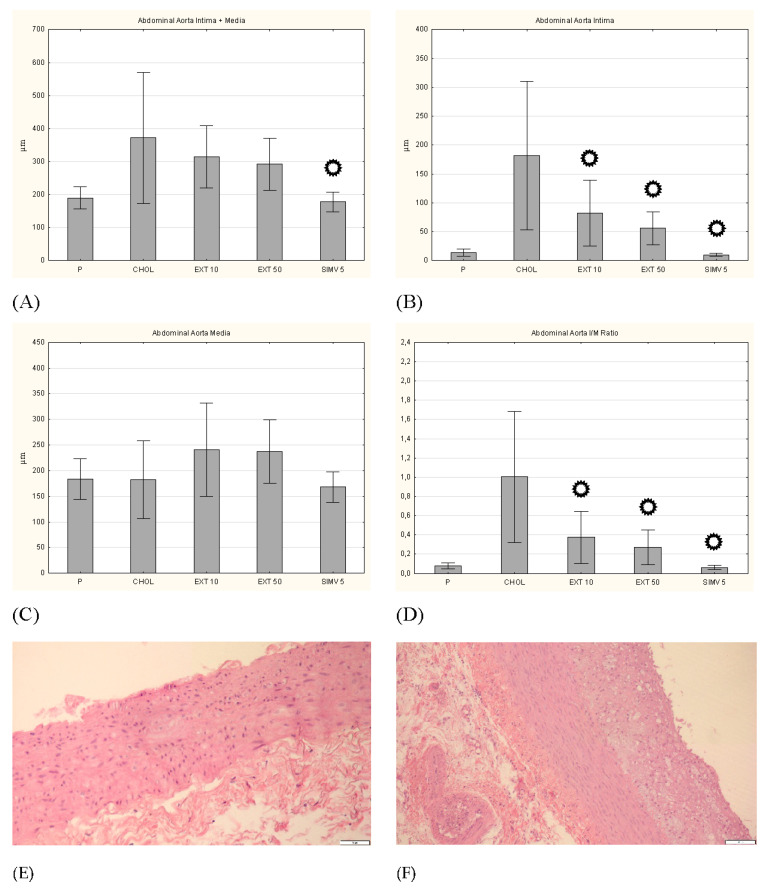

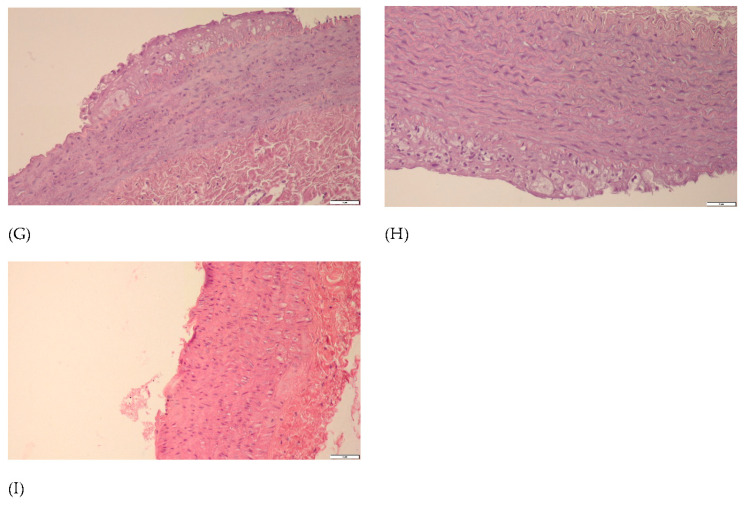
Intima and media thickness, and the intima/media ratio in the abdominal aorta. (**A**) intima + media thickness, (**B**) intima thickness, (**C**) media thickness, and (**D**) I/M ratio. P–standard chow; CHOL–standard chow + 1% cholesterol; EXT 10–standard chow + 1% cholesterol + cornelian cherry extract 10 mg/kg b.w.; EXT 50–standard chow + 1% cholesterol + cornelian cherry extract 50 mg/kg b.w.; SIMV 5–standard chow + 1% cholesterol + simvastatin 5 mg/kg b.w. Values are presented as mean ± SD. 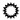 *p* < 0.05 vs. CHOL. Representative cross-sections of the abdominal aorta segments, stained with hematoxylin-eosin in the (**E**) P group, (**F**) CHOL group, (**G**) EXT 10 group, (**H**) EXT 50 group and (**I**) SIMV 5 group.

In the histopathological examination of the thoracic aorta, favorable changes were observed in all groups fed with the assessed compounds, compared to the CHOL group, in which a considerable increase in the diameter of the artery wall was noted. In a complex approach (intima + media), a meaningful reduction in thickness was observed in all three research groups (respectively: *p* = 0.001, *p* = 0.009, and *p* < 0.001). This change particularly pertained to the intima (a significant decrease in all three groups), and to a lesser extent the media (a valuable alteration only in the EXT 10 group). In turn, the measurement of the I/M ratio indicated the EXT 50 (*p* = 0.025) and SIMV 5 (*p* = 0.008) groups as those with the most favorable changes (Figure 7). Moreover, in the histopathological examination of the abdominal aorta, in the collective approach (intima + media), an increase in the wall thickness in the CHOL group and a downward trend in the research groups (the most vivid in the SIMV 5 group–*p* < 0.001) were also observed. The alterations again were particularly visible in the intima assessment (all three groups *p* < 0.001), while in the case of the media, the changes were not statistically meaningful, and in the groups fed with the cornelian cherry extract even an increase in thickness was noted. However, the I/M ratio in both the extract and simvastatin groups showed a significantly positive reduction in value (*p* < 0.001) (Figure 8).

## 4. Discussion

The main conclusion of our study is that oral administration of resin-purified cornelian cherry extract has a positive effect on factors that may contribute to the development of atherosclerosis-related cardiovascular disorders and these effects are at least partially evoked by changes in the expression of transcription factors in the aorta and liver. The key findings are that cornelian cherry extract increases expression of PPAR-α and PPAR-γ in the aorta, and LXR-α expression in hepatocytes. It also decreases the serum levels of leptin, resistin and triglycerides, concomitantly enhancing the level of adiponectin. Moreover, the extract has a positive impact on histopathological changes in the thoracic and abdominal aorta, reducing the I/M ratio. All the observed effects contribute to the limitation of the occurrence of functional and metabolic disorders, which may result in cardiovascular diseases such as atherosclerosis or metabolic syndrome.

As the greatest limitation of our work, we consider the lack of assessment of the extract’s influence on the activity of particularly important target genes for the tested transcription factors. In addition, an increased incidence of cardiovascular diseases and metabolic syndrome is often associated with the Western diet, which apart from being rich in saturated fat and cholesterol, is also characterized by excess carbohydrates and low amounts of fiber. Therefore, the model of animal feeding adopted in our study may not have an unequivocal transfer to the conditions of an actual human diet. Nevertheless, the high-cholesterol feeding allowed us to study the effect of one isolated harmful factor and the possible protective effect of a diet rich in anthocyanins and iridoids as well as provide a basis for further investigations.

Obesity is a condition conducive to the development of diseases of the cardiovascular system. Furthermore, obesity is also associated with an increased risk of all-cause and CVD mortality. Weight reduction is one of the first recommendations and the most important steps to reduce that risk [27,28]. The anthocyanins and iridoids contained in cornelian cherry extract may limit weight gain in a cholesterol-rich diet. Although during the study we observed an increase in body weight in each of the research groups, the gain was to some extent smaller in the EXT 10 and EXT 50 groups. This effect was especially noticeable in the group receiving the higher dose of the extract, where the weight increase was lower by almost 24% compared to the weight increase in the group fed with a cholesterol diet. Weight loss or reduction of weight gain as an effect of iridoids [29,30,31,32] and anthocyanins [33,34,35,36] were also observed in other research models. This also applied to models in which compounds from active extract groups were tested, e.g., loganic acid [37], cyanidin [38] or pelargonidin [39]. What distinguishes our research model from quoted studies, is that it was conducted not on commonly tested rats or mice, but on rabbits–nonrodents that share much more similar lipid metabolic pathways with humans. This may confirm that the positive effect of a diet rich in iridoids and anthocyanins may limit weight gain regardless of species, thereby demonstrating potential efficacy in humans.

Determination of serum lipid levels showed particularly favorable changes in the case of triglycerides. Both in the EXT 10 and EXT 50 groups, there was a statistically significant reduction in the level of TGs compared to the CHOL group. This is highly likely related to the enhanced expression of PPAR-α and PPAR-γ–also demonstrated in our previous studies in the liver [4,22], which activate genes responsible, inter alia, for lipolysis of triglycerides to free fatty acids [40,41]. The elevated concentration of adiponectin in the extract groups may also contribute to this. In turn, in the case of cholesterol levels, both in TC, HDL-C and LDL-C fractions, a certain positive effect of the extract was observed (lowering TC and LDL-C, increasing HDL-C levels), but these changes were relatively small, lesser than observed in the positive control group receiving simvastatin. Other publications reported a more significant effect of iridoids and anthocyanins on plasma/serum cholesterol levels [42,43,44,45,46,47]. However, the qualitative composition of iridoids and anthocyanins in mentioned research models, as well as laboratory animal selection, differed from the composition of the cornelian cherry extract and our animal model.

There are five known transcription factors, that play a crucial role in lipid and cholesterol metabolism: liver X receptor-α, peroxisome proliferator-activated receptor-α, peroxisome proliferator-activated receptor-γ, CCAAT/enhancer-binding protein α (C/EBPα), and sterol regulatory element-binding protein 1c (SREBP-1c) [48]. We assessed the expression levels of PPAR-α and PPAR-γ mRNA in the aorta and LXR-α in hepatocytes.

In both groups fed with the extract, we observed an enhancement in the expression of PPAR-α and PPAR-γ. Interestingly, the increase in expression differed from the dose of the extract used–a dose of 10 mg/kg b.w. caused a greater increase in PPAR-α expression, while the dose of 50 mg/kg b.w. in PPAR-γ. While the increase in PPAR-α expression can rather be evidently associated with a positive effect on cholesterol and lipid metabolism, the effect of PPAR-γ is not so unequivocal. PPAR-α is responsible for the catabolism and intracellular transformations of lipids, through the regulation of the transcription of multiplicitous genes, including acyl-CoA-oxidase, carnitine palmitoyl transferase (CPT) and several CYP4As. PPAR-α is closely related with fatty acid β-oxidation in the peroxisomes and mitochondria, and ω-oxidation in the microsomes. It was also proven that it modulates both acute and chronic inflammation [49,50,51,52,53]. In turn, PPAR-γ plays an important role in the differentiation and maturation of adipocytes, promotes fat storage in white adipose tissue and increases the tissues’ sensitivity to insulin, which leads to a decrease in extracellular glucose levels [49,52,54]. PPAR-γ lowers the transcription of, among others, adipocyte fatty acid-binding protein (aP2), which is responsible for the intracellular binding of fatty acids [55] and lipoprotein lipase (LPL), which regulates the hydrolysis of triglycerides [56,57]. It also enhances the transcription of i.a. fatty acid transporter (FATP), which adjusts transport of fatty acids inside the cells [58], fatty acid-binding protein (L-FABP), which participates in adipogenesis, transport and storage of fatty acids [59]. At the same time, PPAR-γ increases transcription of Acyl-CoA synthetase (lipogenesis and/or catabolism of lipids) [60], ATP-binding cassette G1 (ABCG1), which regulates the transport of cholesterol and phospholipids to macrophages [61], ATP-binding cassette transporter A1 (ABCA1) [62], and also adipocyte-related complement protein 30 (Acrp30), which decrease the concentration of glucose, triclycerides and free fatty acids [40]. Therefore, an enhancement of PPAR-γ expression can cause both positive and negative changes—it all depends on the expression of which of the target genes is activated. In our study, the increase in PPAR-γ expression was accompanied by a mitigation of the intensity of intima/media adverse changes, so it can be associated with a positive vascular effect. We observed a significant reduction in intima diameter in both the thoracic and abdominal aorta. This also translated into favorable I/M ratio results. We can thus hypothesize that the increase in both investigated PPARs expression in the aorta may contribute to limited arterial wall thickening observed in the course of atherosclerosis.

For LXR-α expression, it was the 10 mg/kg b.w. dose that caused a bigger augmentation of expression. This may suggest that a proper dose selection is particularly crucial in achieving the potential full therapeutic effect of the cornelian cherry extract, also in humans. Liver X receptor-α reverses transportation of cholesterol to peripheral tissues and transports the excess cholesterol into the liver, reduces cholesterol absorption by intestinal epithelial cells and directly induces the expression of the essential transcription factor for lipid and cholesterol synthesis in the liver, SREBP-1c [48]. One of the most pivotal target LXR-α genes is ATP-binding cassette transporter A1. LXR-α expression enhance leads to a robust upregulation of ABCA1 in macrophages, intestine, and in the liver and the efflux of cholesterol to apolipoprotein A1 and apolipoprotein E, which results in the formation of high-density lipoproteins (HDL-C) [22,63]. Noteworthy, in the CHOL group there was no increase in LXR-α expression, and even a slight decrease was noted. It seems that the most presumable hypothesis explaining the lack of change in LXR-α expression is that the study period was too brief to observe important alterations. However, since significant changes in LXR-α expression were noted in the test groups, it can be hypothesized that the active compounds contained in cornelian cherry extract activate LXR-α expression in the liver, which has a positive effect on the metabolism of cholesterol.

There are previous reports describing the impact of iridoids and anthocyanins on the expression of transcription factors crucial for cholesterol and lipid homeostasis [48,64]. However, none of these studies concerned the *Cornus mas* L. extract. Moreover, none of the iridoid or anthocyanin surveys have evaluated the changes of PPARs expression in the aorta and their consequences. Considering the results we have obtained, it seems that potentially greater efficacy of the lower dose is a positive result, as it would be more practical for technological development in the form of a drug, e.g., a tablet, which, due to a lesser dose of the active substance, could be smaller and easier for administering. For example: using the formula for dose translation based on BSA [65], calculated dose for adult humans weighing 70 kgs would amount to 227 mg (3.24 mg/kg b.w.), which could be easily compressed in the form of one tablet. As the results of our research are promising, further studies, both determining the dose-effect dependency and conducted in the human model, are necessary.

The adipose tissue secretes several mediators, called adipokines (e.g., adiponectin, leptin, and resistin). Adipokines play an important regulatory function in various metabolic processes and are involved in the development of metabolic diseases, such as type 2 diabetes and cardiovascular diseases. High levels of leptin and resistin occurring in obese individuals, lead to the emergence of insulin resistance, whereas adiponectin may prevent it [66,67]. Leptin is produced and secreted by the adipose tissue in direct relation to the amount of body fat [68]. Circulating leptin positively reflects adipose tissue size, and communicates energy storage status to the brain [69]. We observed a significant reduction of leptin levels in all research groups. The lowering effect of anthocyanins on leptin concentrations was confirmed in many studies [70,71,72,73,74]. Fewer data are available on iridoids. Ishaq et al. hypothesize that asperuloside–iridoid glycoside found in many traditional Chinese medicinal plants–downregulates the gene expression of appetite regulators, i.a. leptin, in the hypothalamus. It overstimulates taste buds signaling in response to high-fat diet consumption leading to suppression of orexigenic signaling [32]. Contrarily, in the case of loganin, an iridoid glycoside isolated from *Cornus officinalis*, an elevation of leptin serum level in type 2 diabetic db/db mice was observed [75]. 

We noted a significant diminution in resistin level in EXT 10 group, in EXT 50 group the effect was lesser. This is in line with our previous findings that a lower dose of the extract could potentially prove to be more effective. There are not many sources describing the effect of the tested compounds on the level of resistin. The reduction of its level in the case of an anthocyanin-rich diet was observed in the rat [67] and mouse model [72]. However, we have not found a study evaluating this effect for iridoids. Resistin was shown to play a pivotal role in various metabolic, inflammatory, and autoimmune diseases [76]. Increased resistin levels are associated with the pathogenesis of obesity-associated insulin resistance and exert pro-inflammatory effects [77,78]. Resistin also appears to mediate the pathogenesis of atherosclerosis by promoting endothelial dysfunction, vascular smooth muscle cell proliferation, arterial inflammation, and the formation of foam cells [79]. Therefore, we can hypothesize that a decrease in the level of resistin together with an increase in PPAR-α and -γ expression translates into the positive effects we observed, limiting adverse changes in the aorta.

Measurement of adiponectin concentration showed the opposite dose–response result. Although the smaller dose augmented the adiponectin level compared to the CHOL group, a significantly beneficial effect of the 50 mg/kg b.w. dose was observed. An enhancement in adiponectin levels was also noted in other studies, evaluating among others asperuloside [80], cyanidin-3-*O*-*β*-glucoside [81], an anthocyanin-rich tart cherry extract comprised mainly of cyanidin glycosides [72] and cornelian cherry extract administered to humans, but only with total anthocyanin-base standardization [82]. An increase in adiponectin quantity is a desirable outcome since adiponectin is important homeostatic factor regulating glucose levels, lipid metabolism, and insulin sensitivity through its anti-inflammatory, anti-fibrotic, and antioxidant effects via two adiponectin receptors, AdipoR1 and AdipoR2 [83]. Circulating adiponectin levels are reduced in obese individuals, and it was proposed to have a crucial role in the pathogenesis of atherosclerosis and cardiovascular diseases associated with obesity and metabolic syndrome. Moreover, adiponectin concentration was found to be correlated with lipoprotein metabolism, especially the high-density lipoproteins and triglycerides. Adiponectin appears to increase HDL-C and decrease TGs levels, among others through activating ATP-binding cassette transporter A1 and lipoprotein lipase and decreasing hepatic lipase [84].

Despite our promising results for adipokines and transcription factors expression, we did not obtain statistically significant outcomes for both glucose and insulin levels. Similarly, the insulin resistance index (HOMA-IR) showed an observable positive trend, but the changes were still in the over normative range. The above findings may hail from species differences resulting from the applicated rabbit model and feeding pattern. However, since the decrease in the value of the HOMA-IR index was, at least in two tested doses, dose-dependent and increasing with its enhancement, it can be hypothesized that the use of a higher dose of the extract may have a more beneficial effect. Although a noticeable increase in HOMA-IR was observed in the CHOL compared to the P group, the feeding schema used (diet enriched with 1% cholesterol) probably also slightly limited the observed effects, because it did not take into account the high level of carbohydrates characteristic for Western diet. The same tendency (dose-dependency) was observed in the case of apolipoproteins, and determination of their levels also requires further research, with various diet models as well as the higher dose or prolonged application of the extract.

The overall positive effect of cornelian cherry extract on detrimental changes induced by a cholesterol-rich diet was confirmed by a decrease in intima and media thickness and the intima/media ratio in the thoracic and abdominal aorta observed in both groups (higher efficacy in the EXT 50 group). Thickening of the arterial wall, especially of the intima layer, is regarded as a risk-marker of atherosclerosis and is described as one of the earliest vascular pathologies observed in microscopic assessment during atherogenesis [85]. Comparable outcomes were obtained by Sozański et al. in another *Cornus mas* fruits study [4,86].

In the end, it is worth noting that in the course of our study we did not observe any adverse effects of cornelian cherry extract administration, regardless of the dose. Therefore, future determinations, including elongated administration or, feasibly, higher doses of the extract, promise a valuable application also in terms of its safety.

## 5. Conclusions

Oral administration of resin-purified cornelian cherry extract showed a diversified contribution to the regression of pathological changes induced by a cholesterol-rich diet in the rabbit model. The positive effects were mainly caused by the increased expression of PPAR-α and PPAR-γ in the aorta and LXR-α expression in hepatocytes, as well as a significant impact on the concentration of adipokines—a decrease in the level of leptin and resistin, and an increase in the level of adiponectin. Additionally, a reduction in the thickness of the thoracic and abdominal aorta walls was observed, and a diminution of the I/M ratio. Our study, to our knowledge, for the first time tested the influence of iridoids and anthocyanins extract on PPARs expression in the aorta, and the rabbit model stood out among the popularly used rats and mice models. The results we have obtained confirm the potential contribution of cornelian cherry extract to mitigate the risk of developing and the intensity of symptoms of obesity-related cardiovascular diseases and metabolic disorders such as atherosclerosis or metabolic syndrome. Thus, cornelian cherry extract may become a valuable resource for reducing the adverse effects caused by the Western diet and constitute a promising pharmaceutical agent. However, further research, especially in humans, is required to fully determine the properties of the extract.

## Figures and Tables

**Table 1:** Experimental groups, feeding plan and scheduled administration of tested substances.

Group	Chow	Tested Substance	Dose of Tested Substance
P	standard chow	none	none
CHOL	standard chow + 1% cholesterol	none	none
EXT 10	standard chow + 1% cholesterol	cornelian cherry extract	10 mg/kg b.w.
EXT 50	standard chow + 1% cholesterol	cornelian cherry extract	50 mg/kg b.w.
SIMV 5	standard chow + 1% cholesterol	simvastatin	5 mg/kg b.w.

**Table 2:** Rabbit average weight on days 0 and 60 and weight change during the study.

Group	Average Weight (kg) [Mean ± SD]		Weight Change (kg)	Weight Change (%)	Change in Weight Gain in Comparison to P Group (%)	Change in Weight Gain in Comparison to CHOL Group (%)
	Day 0	Day 60				
P	3.203 ± 0.338	3.380 ± 0.305	0.177	5.53	0	−57.14
CHOL	3.117 ± 0.453	3.530 ± 0.377	0.413	13.25	133.33	0
EXT 10	3.315 ± 0.320	3.696 ± 0.367	0.381	11.49	115.25	−7.75
EXT 50	3.161 ± 0.434	3.475 ± 0.422	0.314	9.93	77.40	−23.97
SIMV 5	2.975 ± 0.249	3.395 ± 0.274	0.420	14.12	137.29	1.69

**Table 3:** Mean insulin resistance (HOMA-IR) index on day 60.

Group	P	CHOL	EXT 10	EXT 50	SIMV 5
HOMA-IR	2.48	3.58	3.19	2.90	2.73

## Data Availability

The data underlying this article will be shared upon request to the corresponding author.
